# Media Multitasking Is Associated With Higher Body Mass Index in Pre-adolescent Children

**DOI:** 10.3389/fpsyg.2019.02534

**Published:** 2019-11-13

**Authors:** Richard B. Lopez, John Brand, Diane Gilbert-Diamond

**Affiliations:** ^1^Department of Psychology, Bard College, Red Hook, NY, United States; ^2^Department of Epidemiology, Dartmouth College, Hanover, NH, United States

**Keywords:** media multitasking, obesity, body mass index, health risk, adiposity

## Abstract

Obesity rates among children have climbed dramatically in the past two decades, a time period in which children also experienced greater exposure to portable media devices and smartphones. In the present study, we provide evidence of a potential link between media multitasking – using and switching between unrelated forms of digital media – and risk for obesity, as indexed by body mass index (BMI). Specifically, we recruited 179 pre-adolescent children (aged 9–11 years, 88 females) to participate in a study in which we assessed their media multitasking (MMT) tendencies, as well as BMI. Controlling for the influence of a known genetic risk factor for obesity and other covariates, including physical activity, we found a positive association between the frequency of children’s MMT behaviors and age- and sex-standardized BMI *z*-scores, *b* = 1.07, *p* = 0.011. These findings are consistent with other recent work showing similar patterns of covariation between MMT and risk for obesity in young adults. The present work can also inform future work in this realm, such as the design of longitudinal studies that prospectively measure children’s MMT behaviors and body composition to begin to identify directionality in the association.

## Introduction

For much of the 20th century, obesity in childhood was rare, with only 15% of children aged 2–19 classified as overweight or obese in the 1970s. However, by 2004 the childhood obesity rate climbed to nearly 34% and since then has remained high (Fryar et al., 2014; Ogden et al., 2014). Understanding which factors contribute to weight gain early in life is critical, as childhood obesity and overweight have been associated with increased risk for a host of medical conditions, including cardiovascular disease and metabolic syndrome, among others (Must and Strauss, 1999; Dietz, 2004; Daniels et al., 2005; Freedman et al., 2007).

Many genetic and physiological variables contribute to an individual’s risk for developing obesity (e.g., Frayling et al., 2007), as well as dys-regulation of endogenous, homeostatic mechanisms (e.g., Monteleone et al., 2008). For example, the *FTO* rs9939609 single nucleotide polymorphism genotype has been reliably linked to increased obesity risk in large scale studies (Frayling et al., 2007; Loos and Yeo, 2014). But in our modern, 21st-century environment, hedonic features, such as the palatability of foods, can exert a strong influence on eating behaviors, especially among children. This is compounded by the fact that foods, especially obesogenic foods, have become readily available for consumption and are often much less expensive than healthier, nutrient-rich alternatives. Moreover, marketing agencies have targeted children by tailoring food advertisements to increase appeal among children (Bernhardt et al., 2013), and exposure to advertisements has been linked to BMI percentile (McClure et al., 2013) as well as *ad libitum* eating in the absence of hunger (Gilbert-Diamond et al., 2017) – a known correlate of weight status and weight gain (Birch et al., 2003; Lansigan et al., 2015; Balantekin et al., 2016). In general, such exposure to tempting food cues has been associated with increased impulse strength and higher likelihood of un-regulated eating and self-control failure (Heatherton and Wagner, 2011).

Despite these demonstrated links between exposure to appetitive cues and obesity, a key question is: what makes *some* children (and not others) more sensitive and responsive to food cues in their environment? In the present study, we took an individual differences approach by operationalizing, on a person-by-person basis, children’s responsiveness to food cues. To do this, we first drew from Stanley Schachter’s theorizing about the relative influence of internal (e.g., hunger cues) versus external (e.g., the sight or smell of freshly prepared food) cues on eating behaviors. Schacter’s externality theory proposed that individuals with obesity are, on average, more externally driven, meaning they are more responsive to environmental cues that trigger food cravings (Schachter, 1971). While Schachter conceptualized these as group-level differences in the obese and non-obese populations, respectively, we wanted to identify a continuous construct that would manifest behaviorally and capture, at least in part, the extent to which children would be (externally) driven by environmental cues (e.g., food cues).

Media multitasking may be one candidate in the behavioral domain, given that it may train attention to be broader and more sensitive to external cues (Cain and Mitroff, 2011). Indeed, a recent study proposed the novel hypothesis that frequent media multitasking, defined as the simultaneous, often mindless switching between unrelated forms of media, is a risk factor for obesity, as it is associated with increased responsiveness to rewarding cues in one’s environment and poorer self-control (Lopez et al., 2019a). The researchers found supporting evidence for this hypothesis, as those participants who more frequently engaged in media multitasking also tended to have increased adiposity and an imbalance of food cue elicited activity in brain systems involved in reward processing and self-control. Specifically, high media multitaskers showed higher activity in the ventral striatum and orbitofrontal cortex, key regions in the mesolimbic dopamine system that support reward-seeking behaviors, including eating (Haber and Knutson, 2010), while simultaneously exhibiting less recruitment of the frontoparietal control network, which has generally been implicated in flexible exertion of self-control (Power et al., 2011), including self-control in the eating domain (Lopez et al., 2017, 2019b).

In the above-mentioned study by Lopez et al. (2019a), media multitasking tendencies were assessed in college-aged participants, but what about media multitasking habits among developing children and adolescents? Multitasking across several, unrelated forms of media or devices has become common in childhood. Children’s ownership of diverse media devices is increasing. For example, ownership of a cell phone went from fewer than 40% of children in 2004 to 66% by 2009, and the use of other electronic devices (e.g., iPods) increased from 18 to 76% during that period (Rideout et al., 2010). With this rise in device ownership has been a concurrent rise in media multi-tasking; from 1999 to 2009 children nearly doubled their time spent media multi-tasking, from 16% of the time to 29% of the time that they were using media (Rideout et al., 2010). Critically, this trend shows no signs of slowing. From 2011 to 2013, children’s access to mobile devices increased from 52 to 75% (Rideout, 2013), and according to a recent PEW study conducted in 2018, 95% of teens have ready access to a smartphone and 45% report being online almost constantly, up from 24% only a few years prior (Pew Research Center, 2018).

Given the high prevalence of media multi-tasking in youth, as well as the associations observed between multi-tasking and risk of overweight in older populations, we sought to examine the association between children’s media multitasking tendencies and adiposity. Specifically, we conducted a study in which we recruited 179 pre-adolescent children and assessed their media multitasking tendencies and body mass index (BMI). We also controlled for participants’ *FTO* rs9939609 single nucleotide polymorphism genotype, a known genetic risk factor (Frayling et al., 2007; Loos and Yeo, 2014). Given the previous related work that has specifically linked MMT and obesity risk (Lopez et al., 2019a), we hypothesized that, in both unadjusted and adjusted regression models, there would be a positive association between frequency of media multitasking behaviors and BMI.

## Materials and Methods

### Participants

Participants were 198 pre-adolescent children who had taken part in a study that examined the link between an *FTO* polymorphism and food consumption during an eating in the absence of hunger (EAH) task (Gilbert-Diamond et al., 2017). Some of the children who participated in this study were siblings. To ensure the assumption of independence was met for statistical analyses, we included only one randomly selected sibling per family chosen at random. The final sample used for all subsequent analyses consisted of 179 participants (88 females, *M*_age_ = 9.93 years, *SD*_age_ = 0.580 years; see Table 1 for additional summary statistics). As per inclusion criteria determined by that study, all participants had to be fluent in English, present with no food allergies or restrictions, and not have any health conditions nor be taking any medication that would impact appetite or attention span (as reported by participants’ parents). A caregiver accompanied each child who participated, and caregivers and children provided written consent and assent, respectively, in accordance with guidelines set by the Committee for the Protection of Human Subjects at Dartmouth College.

**TABLE 1:** Participant characteristics of sample used in all reported analyses.

	***n* or *M***	**% or *SD***
**Gender**		
Male	91	51%
Female	88	49%
Age	9.93	0.58
***FTO* Genotype**		
TT	63	35%
AT	86	48%
AA	29	16%
MMT score (untransformed)	0.53	0.68
**Weight status**		
Healthy weight	138	77%
Overweight/Obese	41	23%

*Weight status was determined by BMI percentile guidelines set by the CDC, whereby Healthy Weight ≤ 85th and Overweight/Obese > 85th BMI-age-sex-percentile.*

### Procedure

#### Media Multitasking Assessment

One of the most commonly used instruments to comprehensively assess media multitasking tendencies is the Media Multitasking Inventory (MMI; Ophir et al., 2009), but this scale was originally designed to be administered to adults, who sometimes find it cumbersome to complete, given multiple instances of having to estimate hours spent multitasking with various other media (Lopez et al., 2019a). Here, we adapted and shortened the MMI so it would be more easily administered in – and more applicable to – a younger population.

Specifically, we asked participants to report their own multitasking with other print and digital media during four primary activities (versus ten in the original MMI), namely: watching TV or movies, playing video games, reading books or magazines (not assigned for school), and doing homework. There were multiple response options for time spent, daily, for each of these activities (i.e., 0 h, 30 min, 1 h, 1.5 h, 2 h, etc., up to 5 h). For each activity, participants also reported the frequency with which they multitasked by engaging in other activities by using a 5-point likert scale (i.e., *Never*, *Rarely, Sometimes, Often, Always*). For example, after reporting the number of hours spent watching TV and movies, participants also responded to the question “*While you are watching TV and movies, how often do you also play video games or online games at the same time*?” followed by questions to assess multitasking with three additional activities, respectively. This question structure was repeated for each primary activity.

In this way, the kinds of behavior that the MMT measure intends to capture is the simultaneous use of secondary, unrelated media while one uses a primary medium or is engaged in a focal task (e.g., being distracted and checking one’s phone while doing homework). Therefore, it is a different kind of behavior than if a person consciously decides to take a break from an activity. Media multitasking is also different from being in an environment in which media are available or turned on, but one does not seek nor interact with them (e.g., doing homework in one room while the TV happens to be on in the other room). Listening to music was not assessed in the present study because we figured that some participants may be able to focus more intently on the primary task/medium when listening to music, while others would potentially be distracted by it. Thus, the media multitasking score for listening to music would not be straightforward to interpret and combine with other media multitasking scores.

We should note that another research group has encountered the same issues with original MMI as we do here (i.e., the scale’s length and difficulty due to estimating many instances of various primary/secondary media multitasking), and accordingly has shortened and adapted the MMI for use with older adolescents (i.e., ages 11–15; Baumgartner et al., 2014, 2016). These scales (e.g., the short media multitasking measure, or MMM-S; Baumgartner et al., 2016) were not yet published or available when we conducted the present study, but the format and questions overlap considerably because in both cases the items and their wording were taken and adapted from the same source (the original MMI). Moreover, work by Baumgartner et al. (2014) has shown that these shortened scales have high internal reliability in large samples (Cronbach’s alpha = 0.93 in sample of 523) and correlate well with the original MMI (*r* = 0.84; Baumgartner et al., 2016), while being easier and more efficient to administer to adolescents than the original MMI. For all question wording and items included in the abbreviated MMI administered here, see Supplementary  Materials.

Media multitasking scores were calculated using a similar procedure described by Ophir et al. (2009). The total time spent on each activity was multiplied by the frequency of multitasking with other media and then scaling that by the total time spent on all (four) activities (Ophir et al., 2009). This computation was repeated for all four activities, resulting in four MMT sub-scores (one for each activity). These were then summed to create a composite MMT index for each participant (*M*_index_ = 0.53 *SD*_index_ = 0.68, range = 0–3). The distribution of MMT scores was highly positively skewed (γ_1_ = 1.53), so we applied a log transformation with the log10() function from the *base* R package. The resulting distribution exhibited reduced skewness (γ_1_ = 0.85).

#### Body Mass Index

We measured participants’ weight to the nearest 0.1 kg and height to the nearest 0.5 cm using a digital scale (Model 703, Seca, Hamburg, Germany) and wall-mounted stadiometer (Model 216, Seca, Hamburg, Germany). We used these measurements to compute BMI percentile and *z*-score using U.S. Center for Disease Control (CDC) 2000 age- and sex-specific distributions (Kuczmarski et al., 2002). Using the BMI percentiles, we defined healthy weight as ≤85th percentile, overweight as between the 85th and 95th percentile, and obese as ≥95th percentile. We opted to use age- and sex-standardized BMI *z*-score as our outcome of interest, rather than BMI percentile to avoid BMI percentile ceiling effects in the analysis. For age- and sex-standardized BMI scores, we used the y2z() function in the *AGD* (Analysis of Growth Data) R package, with participants’ raw BMI, sex, and age as vectorized inputs, and the CDC 2000 growth curves as the reference table.

#### Covariates

In addition to age and sex, we incorporated all participants’ average physical activity as a covariate into adjusted models by computing a weighted average of time spent (in minutes) being active on weekdays and weekend days, as reported by participants’ caregivers in response to the question(s): “On an average weekday/weekend day, how much time does your child spend doing physical activity, such as running around, climbing, biking, dancing, swimming, playing sports, etc.?”

Importantly, we also controlled for each participant’s genotype status arising from the *FTO* rs9939609 single nucleotide polymorphism to account for the known influence of *FTO* rs9939609 on weight status and risk for obesity (Frayling et al., 2007; Loos and Yeo, 2014). Specifically, buccal cell swabs were collected and stored at room temperature with desiccant capsules (Isohelix, Kent, United Kingdom). DNA was isolated using DDK-50 isolation kits (Isohelix). Genotyping for rs9939609 was conducted with real-time PCR and Taqman chemistry using the 7500 Fast Real-time instrument (primers and instrument from Thermo Fisher Scientific (Waltham, MA, United States). All samples were successfully genotyped and there was 100% genotyping consistency among the 10% blinded replicates.

#### Analytic Approach

We fit both unadjusted and adjusted models with BMI *z*-scores as the outcome measure in order to test for a linear association between MMT and adiposity. In the unadjusted model, log-transformed MMT scores were entered as the sole predictor variable. In the adjusted model, we added participants’ age, sex, *FTO* genotype status, and physical activity as covariates. We indicated a simple contrast for sex (Female > Male) and for *FTO* genotype we indicated a linear contrast to test an additive model of obesity risk (i.e., AA > AT > TT). We also specified an alternative model specification to test the dominant model of obesity risk (i.e., AA and AT > TT) by changing the contrast weights for the *FTO* genotype variable. All regression models were fit using the lm() function from the *stats* R package. To improve interpretation from that model, we then compared BMI *z*-scores between participants with MMT scores at or above the median and participants with MMT scores below the median using unadjusted *t*-tests.

## Results

First, upon examining the unadjusted regression model, there was a significant positive association between log-transformed MMT scores and BMI *z*-score, *r* = 0.181, *b* = 1.037 (95% CI: 0.202, 1.871), *t*(177) = = 2.452, *p* = 0.015. Next, we examined parameter estimates from an adjusted multiple regression model predicting BMI *z*-score as a function of log-transformed MMT scores, while controlling for the potential influence of participants’ age, sex, physical activity, and *FTO* genotype status, as described above. Overall, the model was statistically significant, *F*(6,171) = 4.89, *p* < 0.001, and accounted for 11.6%^1^ of the variance in BMI *z*-scores. There was a statistically significant association between sex and BMI *z*-score, *b* = 0.270 (95% CI: 0.002, 0.538), *t* = 1.99, *p* = 0.048, as well as a statistically significant linear trend in BMI *z*-score as a function of *FTO* genotype status, *b* = 0.494 (95% CI: 0.213, 0.776), *t* = 3.47, *p* = 0.001. Importantly, and central to the aims of the present study, the positive relationship between MMT and BMI *z*-score remained statistically significant in this adjusted model, *b* = 1.069 (95% CI: 0.253, 1.885), *t* = 2.59, *p* = 0.011. This relationship remained unchanged in the sensitivity analysis that included a dominant model of obesity risk as a function of *FTO* genotype, i.e., AA and AT > TT. Moreover, from a model comparison perspective, the AIC was lower and the overall model fit statistically significantly improved when including MMT as a predictor (versus only having age, sex, and *FTO* genotype as predictors), AIC = 472.9 (versus 477.7), *F*(1,172) = 6.68 *p* = 0.011. See Table 2 for complete results from this model, including regression coefficients, confidence intervals, and inferential statistics.

**TABLE 2:** Parameter estimates from a linear regression model predicting BMI *z*-score as a function of log-transformed MMT scores while controlling for age, sex, physical activity, and FTO rs9939609 genotype (additive genetic model: AA > AT > TT).

	**95% confidence interval**					
**Predictor**	**Estimate**	***SE***	**Lower**	**Upper**	***t***	***p***
MMT (log-transformed)	1.069	0.414	0.253	1.885	2.59	0.011
Age in years	−0.154	0.117	−0.385	0.077	−1.32	0.189
Sex (Female > Male)	0.270	0.136	0.002	0.538	1.99	0.048
Physical activity	−0.001	0.001	−0.003	0.001	−1.24	0.215
*FTO* rs9939609 genotype (AA > AT > TT)	0.494	0.143	0.213	0.776	3.47	0.001

Lastly, a median split of the sample based on untransformed MMT scores revealed that those participants who reported relatively more frequent MMT behaviors tended to have higher BMI *z*-scores (*M* = 0.515, *SD* = 1.05) than those with less frequently reported MMT behaviors (*M* = 0.243, *SD* = 0.82); this difference was marginally significant, *t*(177) = 1.90, *d* = 0.285, *p* = 0.059.

## Discussion

In this study, we examined the relationship between children’s propensity to engage in media multitasking behaviors and their risk for higher adiposity. Overall, we found support for our hypothesis, as both unadjusted and adjusted regression models revealed a modest but statistically significant positive relationship between frequency of MMT behaviors and adiposity, as indicated by BMI *z*-score, such that those children who reported more frequent media multitasking also tended to have higher adiposity, on average. These findings are consistent with other work showing similar associations in young adults (Lopez et al., 2019a). Critically, this positive relation between MMT and sex and age-adjusted BMI *z*-score held in an adjusted regression model, suggesting unique co-variation above and beyond the influences of sex, age, physical activity, and *FTO* rs9939609 status – one of the genetic factors most robustly associated with obesity (Frayling et al., 2007; Loos and Yeo, 2014).

Although preliminary, the association observed in this sample between MMT tendencies and adiposity is important to demonstrate, especially in the pediatric population – a group for whom the correlates of media use and media multitasking are not yet well established. In general, children show a pattern in their neurobiological development whereby subcortical systems that support motivated behaviors (e.g., eating) develop early, while maturation of prefrontal systems important for self-control lags behind; this imbalance is especially prominent leading up to and during adolescence (Somerville and Casey, 2010). Because of this imbalance of brain systems, children and adolescents are prone to experience self-control failure and engage in various risk-taking behaviors. It is possible that media multitasking may exacerbate or exploit this imbalance, rendering those children who frequently media multitask unable to exert control over desires to eat that are elicited by tempting food cues in their environment. This is true of young adults, as those who frequently engage in MMT behaviors also tend to show an imbalance in response to appetizing food cues, characterized by more reward related brain activity and less recruitment of the frontoparietal control network (Lopez et al., 2019a), which has been generally implicated in cognitive control as well as self-regulation of eating behaviors (Power et al., 2011; Lopez et al., 2017; Turner et al., 2018). Co-occurrence of such an imbalance and (frequent) media multitasking may arise from biased cognitive and/or attentional processes shaped by children’s exposure to multiple media devices. For example, children who habitually use and switch between multiple, unrelated media may widen their attentional spotlight (Cain and Mitroff, 2011) and this can potentiate reward-related attentional capture (Anderson et al., 2011). This could result in altered global processing of other cues in one’s environment and guide behaviors across domains; indeed, frequent media multitaskers incorporate irrelevant, peripheral cues during person perception, as indicated by biased judgments when forming first impressions (Lopez et al., 2018). This is further consistent with the finding that high media multi-taskers are known to have a greater spread of attention to peripheral cues (Yap and Lim, 2013).

Although the present study supports that media multitasking may be a risk factor for obesity among children, there are several limitations that bear mentioning. First, the associations here are only measured at one point in time, so the directionality of the association cannot be established. As discussed above, it may be that increased exposure to multiple media, and subsequent MMT behaviors, impacts cognition and attention in such a way that some children become more sensitized and responsive to rewarding cues in their environment, increasing hedonically driven consumption. However, alternative explanations are also possible, as it could be that some children have altered cognitive processes and functioning to begin with, and that makes them more likely to become frequent media multitaskers and to be more externally driven by food cues so that non-homeostatic eating patterns result in weight gain over time. Future research is needed to tease apart and test these competing accounts. For example, longitudinal studies that assess children’s MMT tendencies and weight status over longer periods of time can begin to shed light on whether MMT precedes weight gain, or vice versa. Relatedly, it would be important to assess younger children’s baseline responsiveness to external food cues and determine whether that is predictive of subsequent increases in MMT and/or BMI. This is now possible via a newly developed scale that can be completed by parents of preschool-age children (Masterson et al., 2019).

Future studies that experimentally manipulate MMT, in an acute fashion, and measure effects on attention and cued eating, could also help in the determination of causality. This can be difficult, especially given the trade-off between experimental control and ecological validity, but some studies have begun to administer in-lab paradigms that simulate people’s real world exposure to multiple forms of media and in this way provide a context in which media multitasking can occur (e.g., Segijn et al., 2016; Garaus et al., 2017). Another promising avenue for future investigations is to mitigate the negative impacts MMT on cognition and attention via mindfulness-based attention training. Mindfulness interventions are uniquely promising because they promote the opposite of the automatic-based, poorly filtered attention thought to underlie the negative effects of media multi-tasking. A short-term intervention that has been shown to improve mindfulness in undergraduates (Mrazek et al., 2012) has also been shown to reduce the maladaptive attentional processing associated with media multi-tasking. The study conducted with a large sample of young adults showed that a breath counting task, a validated behavioral index of mindfulness (Levinson et al., 2014), improved performance on a battery of attention tasks – including filtering and distractibility – in both high- and low-usual media multi-taskers, but this effect was greater for high media multi-taskers (Gorman and Green, 2016).

We chose to study pre-adolescent children, because this age represents a critical time in development to characterize children’s MMT tendencies and obesity risk; however, the findings may not necessarily be generalizable to children of other ages. We recommend that future work assess MMT and weight status across several age groups to determine if associations vary by age.

We assessed MMT tendencies in children using an adapted version of the MMI scale developed by Ophir et al. (2009). The original MMI is a validated measure that captures people’s propensity to multitask, but it has only adequate predictive and convergent validity, as far as assessing constructs germane to the present work (e.g., trait self-control; Lopez et al., 2019a), and therefore our adapted MMI may also be limited. Baumgartner et al. (2014) have similarly adapted the MMI for use in older adolescents (ages 11–15) and created a 9-item scale that partly overlapped with items administered in the present study (Baumgartner et al., 2014). This 9-item scale exhibited high internal reliability (Cronbach’s alpha = 0.93) while correlating strongly with the original MMI (*r* = 0.84; Baumgartner et al., 2014). Because this scale was not available at the time we collected data from the present sample, we suggest that future work validates this scale in the younger, pre-adolescent population. Future studies may also benefit from administering a recently developed MMT scale that overlaps – in terms of shared variance – with the original MMI but has more construct and convergent validity in the domain of self-regulation (Lopez et al., 2019a). Indeed, stronger associations between MMT and BMI may be observed with either or both of these newly developed MMT scales, given these scales’ respective high internal reliability (Cronbach’s alpha ≥ 0.86; Trafimow, 2016).

Lastly, we generally caution against overinterpretation of the present findings, as the results reported here represent a secondary analysis and therefore we did not have additional covariates. For example, we did not have a measure of participants’ total media consumption or exposure, as that would help determine the nature of the association between MMT and adiposity over and above general media consumption. And despite controlling for a known genetic risk factor for adiposity (*FTO*), we acknowledge the complexity of obesity and its etiology and encourage future researchers to replicate these results while controlling for additional genetic covariates.

To conclude, we have shown that there is a positive association between pre-adolescent children’s media multitasking behaviors and their risk for obesity, as indexed by BMI *z*-score. Specifically, those children who reported frequently using other, secondary media while using a primary medium/device also tended to have higher adiposity. Although definitive causal inferences cannot be drawn from this work, we believe that demonstrating that such an association exists is important and timely, given the rapid rise in both childhood obesity as well as children’s exposure to and use of multiple media devices. Indeed, it is not yet clear how children’s MMT behaviors might impact other cognitive and behavioral domains beyond the realm of eating. And despite the fact that this research is still in its early days, the present findings and their implications suggest that further research is warranted on media multitasking in relation to childhood obesity.

## Data Availability Statement

The datasets generated for this study can be found in the following OSF project (link: https://osf.io/tzv4r/?view_only=7fc5fb7c9b374bf4a5ae52eb8f0189b4).

## Ethics Statement

The studies involving human participants were reviewed and approved by the Committee for the Protection of Human Subjects, Dartmouth College. Written informed consent to participate in this study was provided by the participants’ legal guardian/next of kin.

## Author Contributions

DG-D designed the study. DG-D and RL contributed to hypotheses and research questions. RL and JB conducted the analyses. RL wrote the initial draft of the manuscript. JB and DG-D provided edits and contributed to the final version of the manuscript.

## Conflict of Interest

The authors declare that the research was conducted in the absence of any commercial or financial relationships that could be construed as a potential conflict of interest.

## Footnotes

1 Adjusted *R*-Squared.

## References

[B1] Anderson B. A. Laurent P. A. Yantis S. (2011). Value-driven attentional capture. *Proc. Natl. Acad. Sci. U.S. A.* 108 10367–10371. 10.1073/pnas.1104047108 21646524 PMC3121816

[B2] Balantekin K. N. Birch L. L. Savage J. S. (2016). Eating in the absence of hunger during childhood predicts self-reported binge eating in adolescence. *Eat. Behav.* 24 7–10. 10.1016/j.eatbeh.2016.11.003 27851989 PMC5258820

[B3] Baumgartner S. E. Lemmens J. S. Weeda W. D. Huizinga M. (2016). Measuring media multitasking. *J. Media Psychol.* 29 1–10. 10.1027/1864-1105/a000167

[B4] Baumgartner S. E. Weeda W. D. van der Heijden L. L. Huizinga M. (2014). The relationship between media multitasking and executive function in early adolescents. *J. Early Adolesc.* 34 1120–1144. 10.1177/0272431614523133

[B5] Bernhardt A. M. Wilking C. Adachi-Mejia A. M. Bergamini E. Marijnissen J. Sargent J. D. (2013). How television fast food marketing aimed at children compares with adult advertisements. *PloS One* 8:e72479. 10.1371/journal.pone.0072479 24015250 PMC3756061

[B6] Birch L. L. Fisher J. O. Davison K. K. (2003). Learning to overeat: maternal use of restrictive feeding practices promotes girls’ eating in the absence of hunger. *Am. J. Clin. Nutr.* 78 215–220. 10.1093/ajcn/78.2.215 12885700 PMC2530927

[B7] Cain M. S. Mitroff S. R. (2011). Distractor filtering in media multitaskers. *Perception* 40 1183–1192. 10.1068/p7017 22308888

[B8] Daniels S. R. Arnett D. K. Eckel R. H. Gidding S. S. Hayman L. L. Kumanyika S. (2005). Overweight in children and adolescents: pathophysiology, consequences, prevention, and treatment. *Circulation* 111 1999–2012. 10.1161/01.cir.0000161369.71722.10 15837955

[B9] Dietz W. H. (2004). Overweight in childhood and adolescence. *New Engl. J. Med.* 350 855–857. 10.1056/nejmp048008 14985480

[B10] Frayling T. M. Timpson N. J. Weedon M. N. Zeggini E. Freathy R. M. Lindgren C. M. (2007). A common variant in the FTO gene is associated with body mass index and predisposes to childhood and adult obesity. *Science* 316 889–894. 17434869 10.1126/science.1141634 PMC2646098

[B11] Freedman D. S. Mei Z. Srinivasan S. R. Berenson G. S. Dietz W. H. (2007). Cardiovascular risk factors and excess adiposity among overweight children and adolescents: the bogalusa heart study. *J. Pediatr.* 150 12–17.e2. 17188605 10.1016/j.jpeds.2006.08.042

[B12] Fryar C. D. Carroll M. D. Ogden C. L. (2014). Prevalence of overweight and obesity among children and adolescents: united states, 1963-1965 through 2011-2012. *Nat. Center Health Stat.*

[B13] Garaus M. Wagner U. Bäck A.-M. (2017). The effect of media multitasking on advertising message effectiveness. *Psychol. Market.* 34 138–156. 10.1002/mar.20980

[B14] Gilbert-Diamond D. Emond J. A. Lansigan R. K. Rapuano K. M. Kelley W. M. Heatherton T. F. (2017). Television food advertisement exposure and FTO rs9939609 genotype in relation to excess consumption in children. *Intern. J. Obesity* 41 23–29. 10.1038/ijo.2016.163 27654143 PMC5209258

[B15] Gorman T. E. Green C. S. (2016). Short-term mindfulness intervention reduces the negative attentional effects associated with heavy media multitasking. *Sci. Rep.* 6:24542. 10.1038/srep24542 27086504 PMC4834474

[B16] Haber S. N. Knutson B. (2010). The reward circuit: linking primate anatomy and human imaging. *Neuropsychopharmacology* 35 4–26. 10.1038/npp.2009.129 19812543 PMC3055449

[B17] Heatherton T. F. Wagner D. D. (2011). Cognitive neuroscience of self-regulation failure. *Trends Cogn. Sci.* 15 132–139. 10.1016/j.tics.2010.12.005 21273114 PMC3062191

[B18] Kuczmarski R. J. Ogden C. L. Guo S. S. Grummer-Strawn L. M. Flegal K. M. Mei Z. (2002). 2000 CDC growth charts for the united states: methods and development. *Vital Health Stat.* 246 1–190. 12043359

[B19] Lansigan R. K. Emond J. A. Gilbert-Diamond D. (2015). Understanding eating in the absence of hunger among young children: a systematic review of existing studies. *Appetite* 85 36–47. 10.1016/j.appet.2014.10.032 25450900 PMC4277716

[B20] Levinson D. B. Stoll E. L. Kindy S. D. Merry H. L. Davidson R. J. (2014). A mind you can count on: validating breath counting as a behavioral measure of mindfulness. *Front. Psychol.* 5:1202. 10.3389/fpsyg.2014.01202 25386148 PMC4208398

[B21] Loos R. J. F. Yeo G. S. H. (2014). The bigger picture of FTO: the first GWAS-identified obesity gene. *Nat. Rev. Endocrinol.* 10 51–61. 10.1038/nrendo.2013.227 24247219 PMC4188449

[B22] Lopez R. B. Chen P.-H. A. Huckins J. F. Hofmann W. Kelley W. M. Heatherton T. F. (2017). A balance of activity in brain control and reward systems predicts self-regulatory outcomes. *Soc. Cogn. Affect. Neurosci.* 12 832–838. 10.1093/scan/nsx004 28158874 PMC5460048

[B23] Lopez R. B. Courtney A. L. Wagner D. D. (2019b). Recruitment of cognitive control regions during effortful self-control is associated with altered brain activity in control and reward systems in dieters during subsequent exposure to food commercials. *PeerJ* 7:e6550. 10.7717/peerj.6550 30842910 PMC6397754

[B24] Lopez R. B. Heatherton T. F. Wagner D. D. (2019a). Media multitasking is associated with higher risk for obesity and increased responsiveness to rewarding food stimuli. *Brain Imag. Behav.* 10.1007/s11682-019-00056-0 [Epub ahead of print]. 30820857

[B25] Lopez R. B. Salinger J. M. Heatherton T. F. Wagner D. D. (2018). Media multitasking is associated with altered processing of incidental, irrelevant cues during person perception. *BMC Psychol.* 6:44. 10.1186/s40359-018-0256-x 30305170 PMC6180433

[B26] Masterson T. D. Gilbert-Diamond D. Lansigan R. K. Kim S. J. Schiffelbein J. E. Emond J. A. (2019). Measurement of external food cue responsiveness in preschool-age children: preliminary evidence for the use of the external food cue responsiveness scale. *Appetite* 139 119–126. 10.1016/j.appet.2019.04.024 31047939 PMC6556134

[B27] McClure A. C. Tanski S. E. Gilbert-Diamond D. Adachi-Mejia A. M. Li Z. Li Z. (2013). Receptivity to television fast-food restaurant marketing and obesity among U.S. Youth. *Am. J. Preven. Med.* 45 560–568. 10.1016/j.amepre.2013.06.011 24139768 PMC3934414

[B28] Monteleone P. Castaldo E. Maj M. (2008). Neuroendocrine dysregulation of food intake in eating disorders. *Regul. Pept.* 149 39–50. 10.1016/j.regpep.2007.10.007 18582958

[B29] Mrazek M. D. Smallwood J. Schooler J. W. (2012). Mindfulness and mind-wandering: finding convergence through opposing constructs. *Emotion* 12 442–448. 10.1037/a0026678 22309719

[B30] Must A. Strauss R. S. (1999). Risks and consequences of childhood and adolescent obesity. *Intern. J. Obesity Relat. Metab. Disord.* 23(Suppl. 2), S2–S11. 10.1038/sj.ijo.0800852 10340798

[B31] Ogden C. L. Carroll M. D. Kit B. K. Flegal K. M. (2014). Prevalence of childhood and adult obesity in the United States, 2011-2012. *JAMA* 311 806–814. 10.1001/jama.2014.732 24570244 PMC4770258

[B32] Ophir E. Nass C. Wagner A. D. (2009). Cognitive control in media multitaskers. *Proc. Natl. Acad. Sci.* 106 15583–15587. 19706386 10.1073/pnas.0903620106 PMC2747164

[B33] Pew Research Center, (2018). *Teens, Social Media & Technology 2018.* Washington, DC: Pew Research Center.

[B34] Power J. D. Cohen A. L. Nelson S. M. Wig G. S. Barnes K. A. Church J. A. (2011). Functional network organization of the human Brain. *Neuron* 72 665–678. 22099467 10.1016/j.neuron.2011.09.006 PMC3222858

[B35] Rideout V. (2013). *Zero to Eight: Children’s Media Use in America, 2013.* Available at: https://www.commonsensemedia.org/research/zero-to-eight-childrens-media-use-in-america-2013

[B36] Rideout V. J. Foehr U. G. Roberts D. F. (2010). *Generation M2: Media in the Lives of 8- to 18-Year-Olds.* Available at: http://eric.ed.gov/?id=ED527859 (accessed on July, 2019).

[B37] Schachter S. (1971). Some extraordinary facts about obese humans and rats. *Am. Psychol.* 26 129–144. 5541215 10.1037/h0030817

[B38] Segijn C. M. Voorveld H. A. M. Smit E. G. (2016). The underlying mechanisms of multiscreening effects. *J. Advert.* 45 391–402.

[B39] Somerville L. H. Casey B. J. (2010). Developmental neurobiology of cognitive control and motivational systems. *Curr. Opin. Neurobiol.* 20 236–241. 10.1016/j.conb.2010.01.006 20167473 PMC3014528

[B40] Trafimow D. (2016). The attenuation of correlation coefficients: a statistical literacy issue. *Teach. Statist.* 38 25–28.

[B41] Turner B. M. Rodriguez C. A. Liu Q. Molloy M. F. Hoogendijk M. McClure S. M. (2018). On the neural and mechanistic bases of self-control. *Cereb. Cortex* 29 732–750. 10.1093/cercor/bhx355 29373633 PMC8921616

[B42] Yap J. Y. Lim S. W. H. (2013). Media multitasking predicts unitary versus splitting visual focal attention. *J. Cogn. Psychol.* 25 889–902.

