# Supplementary material for: Media Multitasking Is Associated With Higher Body Mass Index in Pre-adolescent Children
Source: Front Psychol. 2019 Nov 13;10:2534. doi: 10.3389/fpsyg.2019.02534 (PMC6863887; doi:10.3389/fpsyg.2019.02534)
Supplement: Supplementary file 1 [file Data_Sheet_1.docx]

Supplementary Materials

**Abbreviated Media Multitasking Inventory (adapted for use in children)**

1. On an average day, how many hours do you spend watching TV or Movies?

1a. While you are watching TV and Movies, how often do you also play video games or online games at the same time?

1b. While you are watching TV and Movies, how often do you also read books or magazines (that aren’t assigned for school) at the same time?

1c. While you are watching TV and Movies, how often do you also do homework at the same time?

2. On an average day, how many hours do you spend playing video games?

2a. While you are playing video games, how often do you also watch TV and Movies at the same time?

2b. While you are playing video games, how often do you also read books or magazines (that aren’t assigned for school) at the same time?

2c. While you are playing video games, how often do you also do homework at the same time?

3. On an average day, how many hours do you spend reading books or magazines that aren’t assigned for school?

3a. While you are reading books or magazines, how often do you also watch TV and Movies at the same time?

3b. While you are reading books or magazines, how often do you also play video games or online games at the same time?

3c. While you are reading books or magazines, how often do you also do homework at the same time?

4. On an average day, how many hours do you spend doing homework?

4a. While you are doing homework, how often do you also watch TV and Movies at the same time?

4b. While you are doing homework, how often do you also play video games or online games that are not assigned as homework at the same time?

4c. While you are doing homework, how often do you also read books or magazines (that aren’t assigned for school) at the same time?

Eleven options for how much time spent on primary medium:

*0 hr, 30 min, 1 hr, 1 hr 30 min, 2 hrs, 2 hrs 30 min, 3 hrs, 3 hrs 30 min, 4 hrs, 4 hrs 30 min, 5 hr*

Five-point likert scale for multitasking with other digital/print media:

*Never, Rarely, Sometimes, Often, Always*
